# Supplementary material for: Malaria treatment health seeking behaviors among international students at the University of Ghana Legon
Source: PLoS One. 2023 Oct 26;18(10):e0276412. doi: 10.1371/journal.pone.0276412 (PMC10602253; doi:10.1371/journal.pone.0276412)
Supplement: Attachment — Submitted filename: PONE-D-22-27540_malaria.pdf [file pone.0276412.s003.pdf]

PLOS ONE

Malaria treatment health-seeking behaviors among international students at the University of Ghana Legon, Accra

--Manuscript Draft--

Manuscript Number: PONE-D-22-27540

Article Type: Research Article

Full Title: Malaria treatment health-seeking behaviors among international students at the University of Ghana Legon, Accra

Short Title: Malaria treatment health-seeking behaviors among international students at University of Ghana Legon, Accra

Corresponding Author: Mathias Lwenge, MSc. Applied Health Social Sciences
Uganda Martyrs University
Kampala, UGANDA

Keywords: Malaria; treatment seeking behavior; Ghana; international students

Abstract:

Introduction

Appropriate malaria management necessitates early health-seeking behavior upon suspicion of malaria-like symptoms. This study looked at malaria treatment-seeking behavior and the factors that influence it among international students at the University of Ghana. Appropriate malaria health-seeking behavior was defined as an international student experiencing symptoms suggestive of malaria and eventually visiting a health facility where appropriate management, including a blood test to confirm malaria, would be carried out, followed by appropriate malaria treatment.

Methods

This is a quantitative cross-sectional study that collected data from 264 international students on malaria treatment-seeking behavior and factors influencing this behavior. The Pearson Chi-square (X2) test of independence was used to assess associations between individual characteristics and Malaria treatment-seeking behavior. The binary logistic regression model was constructed using a backward Wald method, with variables retained at a Wald p-value of 0.05.

Results

According to the findings, 35% of respondents obtained self-prescribed antimalarials as their first choice of Malaria treatment from a pharmacy. A significant relationship exists at the bivariate level between appropriate malaria health-care seeking behavior and: - Respondents continent, X2(1, N = 264) = 7.936, p =.005; Service accessibility, X2(1, N = 264) = 7.624, p =.006; Wait time, X2(1, N = 264) = 22.514, p< 0.001; Treatment cost, X2(1, N = 264) = 97.160, p <0.001; Health insurance, X2(1, N = 264) = 5.837, p <0.001. In a multivariable analysis, inappropriate malaria health-seeking behaviors were associated with low perceived service accessibility (330mins) (aOR = 6.67; p<0.001), perceived long wait time (330mins) (aOR = 5.94; p=0.015), perceived treatment cost affordability (15<GHC) (aOR = 19.88; p<0.001), and age group: 34-41years (aOR = 8.83; p<0.001).

Conclusion

There was widespread inappropriate health-care-seeking behavior for Malaria treatment among international students. Improving access to malaria treatment services, as well as lowering wait times and treatment costs, will address inappropriate malaria treatment health-seeking behavior among international students.

Order of Authors:

Mathias Lwenge, MSc. Applied Health Social Sciences

Philip Govule

Simon Peter Katongole

Powered by Editorial Manager® and ProduXion Manager® from Aries Systems Corporation

	Phyllis Dako-Gyeke
**Opposed Reviewers:**	
**Additional Information:**	
**Question**	**Response**
**Financial Disclosure**<br><br>Enter a financial disclosure statement that describes the sources of funding for the work included in this submission. Review the submission guidelines for detailed requirements. View published research articles from *PLOS ONE* for specific examples.<br><br>This statement is required for submission and **will appear in the published article** if the submission is accepted. Please make sure it is accurate.<br><br>**Unfunded studies**<br>Enter: *The author(s) received no specific funding for this work.*<br><br>**Funded studies**<br>Enter a statement with the following details:<br>• Initials of the authors who received each award<br>• Grant numbers awarded to each author<br>• The full name of each funder<br>• URL of each funder website<br>• Did the sponsors or funders play any role in the study design, data collection and analysis, decision to publish, or preparation of the manuscript?<br>• **NO** - Include this sentence at the end of your statement: *The funders had no role in study design, data collection and analysis, decision to publish, or preparation of the manuscript.*<br>• **YES** - Specify the role(s) played.<br><br>* typeset	Mathias Iwenge (ML)<br>Simon Peter Katongole (SPK)<br>Philip Govule (PG)<br>Phyllis Dako Gyeke (PDG)<br>ML and PD-G initiated the project. ML conducted the data analysis with considerable input from PG. ML was involved in development of first draft of the manuscript with PG, SPK, and reviewed by PD-G. All authors contributed to the subsequent versions of the manuscript
**Competing Interests**<br><br>Use the instructions below to enter a competing interest statement for this submission. On behalf of all authors, disclose any competing interests that could be perceived to bias this work—acknowledging all financial support and any other relevant financial or non-	The authors have no conflicts of interest.

<table>
<tr><td>financial competing interests.

This statement is required for submission and will appear in the published article if the submission is accepted. Please make sure it is accurate and that any funding sources listed in your Funding Information later in the submission form are also declared in your Financial Disclosure statement.

View published research articles from PLOS ONE for specific examples.

NO authors have competing interests

Enter: The authors have declared that no competing interests exist.

Authors with competing interests

Enter competing interest details beginning with this statement:

I have read the journal's policy and the authors of this manuscript have the following competing interests: [insert competing interests here]

* typeset</td><td></td></tr>
<tr><td>Ethics Statement

Enter an ethics statement for this submission. This statement is required if the study involved:

• Human participants
• Human specimens or tissue
• Vertebrate animals or cephalopods
• Vertebrate embryos or tissues
• Field research

Write "N/A" if the submission does not require an ethics statement.

General guidance is provided below.
Consult the submission guidelines for</td><td>Ethics approval, procedures and consent to participate in the study
Ghana Health Service Ethics Review Committee granted ethical approval to conduct the study (Approval No: GHS-ERC023/12/17) (GHS-ERC). Permission was granted by the Dean of the International Program Office, the Dean of Students, and the Senior Hall Tutor for Jubilee Hall and the International Students' Hostel at the University of Ghana where the international students reside. All the participants provided written informed consent. There are no personal identifiers on the tools or analysis</td></tr>
</table>

Powered by Editorial Manager® and ProduXion Manager® from Aries Systems Corporation

detailed instructions. **Make sure that all information entered here is included in the Methods section of the manuscript.**<br><br>**Format for specific study types**<br><br>**Human Subject Research (involving human participants and/or tissue)**<br>• Give the name of the institutional review board or ethics committee that approved the study<br>• Include the approval number and/or a statement indicating approval of this research<br>• Indicate the form of consent obtained (written/oral) or the reason that consent was not obtained (e.g. the data were analyzed anonymously)<br><br>**Animal Research (involving vertebrate animals, embryos or tissues)**<br>• Provide the name of the Institutional Animal Care and Use Committee (IACUC) or other relevant ethics board that reviewed the study protocol, and indicate whether they approved this research or granted a formal waiver of ethical approval<br>• Include an approval number if one was obtained<br>• If the study involved *non-human primates*, add *additional details* about animal welfare and steps taken to ameliorate suffering<br>• If anesthesia, euthanasia, or any kind of animal sacrifice is part of the study, include briefly which substances and/or methods were applied<br><br>**Field Research**<br><br>Include the following details if this study involves the collection of plant, animal, or other materials from a natural setting:<br>• Field permit number<br>• Name of the institution or relevant body that granted permission	
**Data Availability**<br><br>Authors are required to make all data underlying the findings described fully available, without restriction, and from the time of publication. PLOS allows rare	Yes - all data are fully available without restriction

exceptions to address legal and ethical concerns. See the PLOS Data Policy and FAQ for detailed information.<br><br>A Data Availability Statement describing where the data can be found is required at submission. Your answers to this question constitute the Data Availability Statement and **will be published in the article**, if accepted.<br><br>**Important:** Stating 'data available on request from the author' is not sufficient. If your data are only available upon request, select 'No' for the first question and explain your exceptional situation in the text box.<br><br>Do the authors confirm that all data underlying the findings described in their manuscript are fully available without restriction?	
**Describe where the data may be found in full sentences. If you are copying our sample text, replace any instances of XXX with the appropriate details.**<br><br>• If the data are **held or will be held in a public repository**, include URLs, accession numbers or DOIs. If this information will only be available after acceptance, indicate this by ticking the box below. For example: *All XXX files are available from the XXX database (accession number(s) XXX, XXX.*).<br>• If the data are all contained **within the manuscript and/or Supporting Information files**, enter the following: *All relevant data are within the manuscript and its Supporting Information files.*<br>• If neither of these applies but you are able to provide **details of access elsewhere**, with or without limitations, please do so. For example:<br><br>*Data cannot be shared publicly because of [XXX]. Data are available from the XXX Institutional Data Access / Ethics Committee (contact via XXX) for researchers who meet the criteria for*	All relevant Data will be provided upon request by the editors upon request and for the researchers who meet the criteria of access to confidential data

access to confidential data.<br><br>*The data underlying the results presented in the study are available from (include the name of the third party and contact information or URL).*<br>• This text is appropriate if the data are owned by a third party and authors do not have permission to share the data.<br><br>* typeset	
Additional data availability information:	

Powered by Editorial Manager® and ProduXion Manager® from Aries Systems Corporation

Cover letter

Mathias Lwenge
P.O BOX, 5498,
Kampala, Uganda
Date 24th /09/2022

The Editor in Chief
PLOS ONE Journal
Dear sir/madam

I hereby submit to the PLoS ONE Journal this manuscript titled "Malaria treatment health-seeking behaviors among international students at the University of Ghana Legon, Accra." This is an original research paper derived from the corresponding author's Master of Science dissertation, which was undertaken in Ghana. A quantitative cross-sectional study was conducted at the University of Ghana to investigate malaria treatment-seeking behavior and the factors that influence it.

Readers of this journal will be interested in reading this article because students have been observed to exhibit unusual health-seeking behaviors when they become ill, and with malaria being one of the potential diseases they are at risk of contracting during their stay in a malaria-endemic country, it is necessary to understand how they would act if they developed signs and symptoms suggestive of malaria. Unfortunately, most research on malaria health seeking behaviors has primarily focused on travelers, with information among students who are likely to stay in host countries for a longer period of time scarce. This is against the backdrop of an increase in the number of foreign students seeking to study in tropical countries.

The findings of this study may help universities determine how to best manage students suspected of having malaria while studying in tropical countries. International students planning to study in malaria-endemic countries should be aware of the potential challenges they will face as they navigate their study countries if they become ill with malaria-like symptoms.

We followed all the ethical principles outlined by the Ghana Health Service Ethics Review Committee, as detailed in the manuscript. We confirm that this manuscript has not been previously published and is not being considered by another journal. The manuscript has been approved by all authors, to be submitted to the PLoS ONE journal.

We will be delighted if this manuscript is accepted for publication after a thorough peer review, as it will help advance the academic careers of some of the authors, who are early career researchers.

Sincerely, yours

Mathias Lwenge mathiaslwenge@gmail.com

Corresponding author

Manuscript

Click here to access/download;Manuscript;Malaria_Manuscript__Lwenge_Mat

Malaria treatment health-seeking behaviors among international students at the University of Ghana Legon, Accra

Authors: Mathias Lwenge[1,5] *, Philip Govule[2,3]*, Simon Peter Katongole[4], Phyllis Dako-Gyeke[1]

Affiliations:

[1]Department of Social and Behavioural Sciences, College of Health Sciences, School of Public Health, University of Ghana, Ghana

[2]Department of Epidemiology and Disease Control, College of Health Sciences, School of Public Health -University of Ghana, Ghana

[3]Department of Government studies, School of Management Studies, Uganda Management Institute, Uganda

[4]Department of Health Policy, College of Health Sciences, School of Public Health, University of Ghana, Ghana

[5] Faculty of Health Sciences , Uganda Martyrs University, Uganda

Email address of author :ML,mathiaslwenge@gmail.com, SPK spkatongole@gmail.com , PG pgovule@gmail.com, PDG gyekenay@yahoo.com

Keywords:

Malaria, treatment seeking behavior, Ghana, international students

ABSTRACT

Introduction: Appropriate malaria management necessitates early health-seeking behavior upon suspicion of malaria-like symptoms. This study looked at malaria treatment-seeking behavior and the factors that influence it among international students at the University of Ghana. Appropriate malaria health-seeking behavior was defined as an international student experiencing symptoms suggestive of malaria and eventually visiting a health facility where appropriate management, including a blood test to confirm malaria, would be carried out, followed by appropriate malaria treatment.

Methods: This is a quantitative cross-sectional study that collected data from 264 international students on malaria treatment-seeking behavior and factors influencing this behavior. The Pearson Chi-square (X2) test of independence was used to assess associations between individual characteristics and Malaria treatment-seeking behavior. The binary logistic regression model was constructed using a backward Wald method, with variables retained at a Wald p-value of 0.05.

Results: According to the findings, 35% of respondents obtained self-prescribed antimalarials as their first choice of Malaria treatment from a pharmacy. A significant relationship exists at the bivariate level between appropriate malaria health-care seeking behavior and: - Respondents continent, $X^2(1, N = 264) = 7.936$, p =.005; Service accessibility, $X^2(1, N = 264) = 7.624$, p =.006; Wait time, $X^2(1, N = 264) = 22.514$, p< 0.001; Treatment cost, $X^2(1, N = 264) = 97.160$, p <0.001; Health insurance, $X^2(1, N = 264) = 5.837$, p <0.001. In a multivariable analysis, inappropriate malaria health-seeking behaviors were associated with low perceived service accessibility (^{3}30mins) (aOR = 6.67; p<0.001), perceived long wait time (^{3}30mins) (aOR = 5.94; p=0.015), perceived treatment cost affordability (15<GHC) (aOR = 19.88; p<0.001), and age group: 34-41years (aOR = 8.83; p<0.001).

Conclusion: There was widespread inappropriate health-care-seeking behavior for Malaria treatment among international students. Improving access to malaria treatment services, as well as lowering wait times and treatment costs, will address inappropriate malaria treatment health-seeking behavior among international students.

Introduction

Healthcare seeking behaviour is any action or inaction taken by individuals who believe they have a health problem or are ill in order to find proper medication (1). Malaria is one of the illnesses that calls for appropriate healthcare seeking behaviours among individuals(2). Despite extensive efforts to control it, malaria remains a major public health challenge around the world (3). In 92 countries, approximately 1.1 billion people are considered to be at high risk of contracting malaria (4). Malaria was prevalent in approximately 85 countries by 2020, with 241 million cases reported globally and 627 000 deaths (5). Sub-Saharan Africa bears the greatest burden of malaria worldwide, accounting for 95% of the prevalence and 93% of malaria deaths (6). Malaria is also the most common communicable disease seen in Sub-Saharan Africa's health facilities (7). With over 300 million people at risk of malaria infection, the Western Africa sub-region accounts for roughly half of the global Malaria burden(2). Malaria is still endemic in Ghana, accounting for 2% and 3% of global malaria cases and deaths, respectively (8). As a result, Ghana is one of the top 15 countries in the world with a high malaria burden (5). Malaria accounts for up to 32.5% of all outpatient cases in Ghanaian health facilities (9).

The World Health Organization (WHO) recommends early diagnosis and prompt treatment of malaria as a key requirement for effective malaria case management (8). The WHO Global Malaria Control Program introduced and launched the 3Ts (Test, Treat, and Track) initiative in April 2012, aimed at improving drug testing and provision based on results and proper case reporting (10). As a result of the adoption of the "3Ts" strategy, the guidelines for treating malaria were revised and required that suspected malaria cases should be tested and the treatment be determined based on the results of either the Rapid Diagnostic Test (RDT) or microscopy (11). Adhering to this guideline by a patient who suspects they have malaria constitutes appropriate malaria health seeking behavior, which is required for effective malaria case management and prevention (4,5). This is due to the presence of malaria-like symptoms in other febrile conditions besides malaria, necessitating individuals with fever to seek care from a suitable institution to rule out malaria or other causes of fever (12).

Ineffective malaria management due to incorrect decisions may result in delayed diagnosis and treatment, increasing the risk of severe disease and death **Error! Reference source not found.,Error! Reference source not found.**. Factors such as lack of access to healthcare, knowledge and attitude toward malaria treatment, health insurance coverage, and socioeconomic factors may lead to inappropriate malaria health seeking behavior by suspected malaria clients, resulting in poor malaria management (13).

The risk of contracting malaria varies by population. People from non-malaria endemic countries including international students are at a high risk of contracting malaria in malaria endemic countries like Ghana because they have low immunity to malaria due to lacking exposure to the plasmodium (13,14). They must also negotiate various health service access barriers while attempting to obtain appropriate malaria care (14). Similarly, students from malaria-endemic areas may adopt malaria health-seeking behaviors and practices from their home country that differ from those required in Ghana. As a result, such students are likely to receive ineffective malaria treatment. Such students are vulnerable to severe malaria consequences, particularly during peak mosquito breeding season(15)

It is prudent for international students from both endemic and non-endemic malaria countries to adopt malaria prevention and management health seeking behaviors that are consistent with their host countries' guidelines. Although there is sufficient information on the prevalence and treatment behaviors of imported malaria by students returning from high malaria endemic countries in Africa, there is limited evidence on their health seeking behaviors after contracting the disease while living in high malaria endemic countries (16,17). International students account for a sizable proportion of international travelers in Ghana. As a result, the goal of this study was to assess malaria treatment seeking behaviors and associated factors among malaria-susceptible international students at the University of Ghana's four colleges. Obtaining information about their malaria management health seeking behavior and the factors that influence this behavior will help improve malaria management, prevention, and control among international students at universities in malaria endemic countries.

Methods

Study design

A descriptive cross-sectional quantitative study design was used out in the context of the 'actions taken' approach to investigate malaria health-seeking behavior. The approach taken is described as the 'first' and ‘second' lines of action taken by individuals who appear to feel unwell following the development of malaria-related symptoms (18).

Study setting

The University of Ghana is Ghana's leading and oldest university, having opened its doors in 1948. (19). It has two hospitals, a sickbay that provide students with relatively free Primary Health Care (PHC) services. It also has a number of private pharmacies located nearby (19).

Study population

As of July 2018, the study population consisted of 840 international students, accounting for 2.14% of the total University student population of 39,249 students (20).

Sample size and Sampling procedures

The sample size for the study was calculated using the single proportion formula: $n = \frac{Z^2PQ}{\delta^2}$ (21). Where: n is the minimum sample size, z is the standard normal deviation at 95% confidence (1.96), p is the estimated proportion of students with good malaria health seeking behavior, and because the prevalence of health malaria seeking behavior among international students was unknown, a conventional prevalence (P) estimate of 50% was taken as a reasonable estimate of the proportion of international students with good malaria health seeking behavior in Ghana, Q=1-p, δ=desired level of precision (0.05) at 95% CI. As a result, the desired sample size after finite population correction and a 10% non-response rate resulted in a total of 291 students. The study participants were chosen using a systematic sampling technique with a pre-designed sampling frame comprised of registered international students with the university's International Program Office (IPO). Only international students enrolled at the university during the 2017/2018 academic year were considered.

Data Collection procedures and Instruments

A self-administered structured questionnaire was used in a university-based survey conducted between March and June 2018. Data on socio-demographic characteristics, individual enabling characteristics, malaria knowledge, and malaria treatment seeking behavior the last time the participant experienced malaria-like signs and symptoms. Questions on malaria transmission, mosquito breeding sites, signs and symptoms, preventive measures, and malaria information sources were used to assess respondents' level of knowledge. Data collection procedures validated the questionnaire. Each participant's address (phone number and hall of residence) was obtained from the IPO student list. The respondents were summoned for a meeting by the researcher (ML), during which the reasons for conducting the study were explained and consent for participation was obtained.

Study Variables and scale of Measures

The study focused on respondents' Malaria health-care seeking behavior during their most recent illness. The outcome variable was whether an international student at the University of Ghana sought malaria treatment at a health facility for suspected malaria or not (if care was sought elsewhere other than a health facility ~~where malaria testing and management is done for cardinal malaria symptoms such as fever, headache, and joint pains~~). The following query was posed: Where did you go for treatment the last time you had signs and symptoms like fever, joint pains, or a headache? The responses of the respondents were classified into two groups, resulting in a binary variable by coding respondents who sought Malaria treatment from a 'health facility' as defined above into one group and coded as 'Yes =1', while all those who sought care from an alternative location other than a health facility [traditional healers, herbalists, drug shops without

an adequate diagnosis, or those who never sought any treatment despite suspecting Malaria'] were all labeled as a No=0.

The categorical independent variables considered for factors influencing health seeking behavior included: service accessibility (classified as more accessible for those that took less than 30 minutes to reach facility;(less accessible if ≥30mins); waiting time: short (<30mins), long: (≥30mins); affordability: ~~more affordable (<15 Ghana Cedis (GHC) and less affordable~~ (needing to pay ≥15 Ghana Cedis (GHC) to get services; cost of transportation (classified as more affordable for cost of <8GHC centre and less affordable 8GHC to reach services) (categorized as friendly or not friendly). Others included; Health insurance (No, Yes); perceived service availability (No, Yes); Knowledge on Malaria (Knowledgeable, not knowledgeable), and perception of health worker attitude (categorized as friendly or not friendly).

Other considerations included the respondent's residential status (whether a university campus resident or a non-resident), sponsorship status (whether self-sponsored or on a paid scholarship), and ~~health insurance coverage (yes or no).~~ we further considered the cost of malaria treatment during the most recent illness. We also controlled for respondents' socio-demographic characteristics that were treated as categorical variables: Age (categorized as less than 29 years, 30-39 years, 40-49 years, and >50 years). Sex (male or female), marital status (married or single), education level (bachelor's, master's, or doctorate), continent of origin (Africa, other continents), and college of study (Health sciences, basic and applied health sciences, humanities, and college of education) were also factored in.

Data management and analysis

~~Data were checked for completeness and consistency, coded, and entered into a predesigned excel template before being exported to IBM's SPSS statistical software version 22 (V22) for analysis. At the univariate level, descriptive statistics were used to organize and summarize the data as frequencies, proportions for categorical variables, and mean values for continuous variables. In bivariate analysis, the Pearson Chi-square statistics (X^2) were used~~ exclusively to establish the relationship between categorical variables and malaria treatment seeking behavior from a healthcare facility because all variables met the set Pearson X^2 assumptions (eg. Larger cell counts .ie. equal, and above five etc).

To estimate the effects of each predictor or explanatory variable on the outcome variable, univariate and multivariable binary logistic regression analyses were performed. At the univariate analysis, the predictor variable was considered individually, and it was later included with other (more than one) variables in the multivariable model. Explanatory (predictor) variables that were associated with the outcome of interest (malaria treatment-seeking behavior) at a threshold of 0.2 ($p \leq 0.20$) level were included in multivariable regression, and predictor variables with Wald p-value <0.05 were retained in the final multivariable models. These were considered to be independently associated with international students' choice of malaria treatment seeking behavior and documented as adjusted odds ratio (aOR) at 95% confidence interval CI. Hosmer and Lemeshow's goodness of fit test was used to evaluate the final model's fit to the data. Unadjusted measures were extracted at the level of univariate logistic regression analysis when conducting multivariate analysis. The models calculated the odds ratio as a measure of the associations between the outcome and explanatory variables, as well as the 95% confidence interval. We present these findings as crude Odds Ratios (cOR) with 95% confidence intervals (CI). We performed a multicollinearity test prior to multivariable logistic regression analysis by running a multiple linear regression with the goal of excluding collinear variables (variables with a Variance Inflation Factor (VIF) greater than 10). There was no evidence of multicollinearity in the results from any of the explanatory factors.

RESULTS

General characteristics of the respondents

More than half of the respondents (51.1%, 135/264) were men. Participants ranged in age from 18 to 39 years, with a mean age of 25.2 years. More than half of those polled (58.7%, 155/264) were between the ages of 18 and 25years. The majority of respondents (71.2%, 188/264) were from African countries. More than half of the respondents, 54.2%, had valid health insurance. The majority of respondents (87.1%, 230/264) lived on either the university's main campus or the Korle-Bu medical school campus, which houses the College of Health Sciences. The majority of respondents were Christians (77.4%, 204/264), not married or single (79.5%, 210/264. The College of Basic and Applied Health Sciences had the highest proportion of respondents (39.4%) while the College of Education had the least representation (8%).

Malaria treatment health-seeking behaviors

We investigated the respondents' immediate action in terms of seeking treatment as soon as they suspected they had malaria-like symptoms. Table 1 shows detailed cross-tabulations of actions taken to seek Malaria treatment. Five patterns of malaria treatment seeking behavior emerged. These included health seeking behaviors toward orthodox care, such as treatment being facilitated by purchasing medicines from a pharmacy for either antimalarial treatment only or antimalarial and analgesics, or for some purchasing only analgesics. Other non-orthodox malaria treatment seeking behaviors included seeking care at a health facility or using alternative treatments such as herbs, prayers, and warm baths. Over 86.3% of respondents purchased drugs from a pharmacy as their first malaria treatment option, with 35.2% purchasing only anti-malarial drugs, 26.9% purchasing both anti-malarial and analgesics, and 24.2% purchasing both anti-malarial and analgesics while 24.2% bought only analgesic. Only 10 (2.7%) of the 264 respondents went straight to the health facility when they suspected malaria. There was a slight difference in the proportion of males and females who purchased medicines at a pharmacy or drug store as their first treatment option (43.9% and 42.5%, respectively).

Overall, the proportion of African and non-African students who sought treatment from a pharmacy was nearly identical (86.1% for African versus 87.8% for non-African). The majority of non-Africans (42.1%) purchased an anti-malarial as the first line of treatment. Using only an anti-malarial (32.4%) or both an anti-malarial and analgesic (31.9%) purchased from a pharmacy or drug store was common among African international students. Whereas only 10/188 (3.7%) of African international students sought treatment from a health facility as the first point of care for malaria, no non-African international students sought treatment from a health facility as the first point of care for malaria. We also looked into what the next step should be if someone did not improve, including those who had sought care at the health facility but returned for a second opinion. The majority of respondents (57.6%) went to health facilities, while 38.3% sought additional care from a pharmacy or drug store. The proportion of males and females seeking care from a health facility as a second line of treatment for malaria was slightly different (58.5% for males versus 56.6% for females). Non-African international students (71.1% of the 76) sought second-line care from a health facility more than African international students (52.1%).

Table 1: ***Actions taken to seek for health care by sex and their continent of residence (N=264)***

Action	Total (N=264)	Sex		Continent	
		Males	Females	Africa (n=188)	Non-African (n=76)
	N (%)	n(%)	n(%)	n(%)	n(1%)
**The first line of action**					
Antimalarial only from a pharmacy	**93(35.2)**	53(39.3)	40(31%)	61(32.4)	32(42.1)
Antimalarial +painkillers pharmacy	**71(26.9)**	38(28.1)	33(25.6)	60(31.9)	11(15.4)
Painkillers only from a pharmacy	**64(24.2)**	25(18.5)	39(30.2)	41(21.8)	23(30.3)
Went to a health **facility (hospital/clinic**	**10(2.7)**	7(3.7)	3(1.6)	10(3.7)	0(0.00)
Non-medical actions	**26(11)**	12(10.4)	14(11.6)	16(10.2)	10(12.2)
**The second line of action**					
Health facility (hospital/clinic) [a]	**152(57.6)**	79(58.5)	73(56.6)	98(52.1)	54(71.1)
Pharmacy (drug shop)	**101(38.3)**	53(39.3)	48(37.2)	80(42.6)	21(27.6)
Non-medical actions [b]	**11(4.2)**	3(2.2)	8(6.2)	10(5.3)	1(4.2)
**Total**	**264(100)**	**135(100)**	**129(100)**	**188(100)**	**76(100)**

a= Included those who had first sought for care at health facility and returned for review; b=Herbs/ prayers/warm bath

Characteristics of participants stratified by Malaria Treatment Seeking Behaviour

At bivariate analysis, there was a significant relationship between Malaria treatment health-care seeking behavior and respondent continent, X^2 (1, N = 264) = 7.936, p =.005. Respondents from other continents were more likely than Africans to report seeking Malaria treatment appropriately following the most recent febrile illness suspected to be malaria. The relationship between perceived service accessibility and X^2 (1, N=264) =7.624, p =.006. Students who perceived service accessibility as less accessible or 30 minutes were more likely than those who perceived service accessibility as more accessible. There was also a statistically significant relationship between Malaria treatment seeking behavior and perceived waiting time, X^2 (1, N = 264) = 22.514, p < 0.001; respondents who perceived waiting time as long were more likely to report seeking malaria treatment appropriately (from health facility as recommended). The relationship between Malaria treatment seeking behavior and perceived treatment cost was statistically significant, X^2 (1, N= 264) = 97.160, p < 0.001. Respondents who self-reported treatment costs as affordable (15 GHC) were more likely to report seeking appropriate malaria treatment (from health facility).

There was a statistically significant relationship between Malaria treatment seeking behavior and report of having health insurance cover, X^2 (1, N= 264) = 5.837, p = 0.016; respondents who self-reported as not being covered by health insurance were more likely to report themselves to have sought malaria treatment appropriately (from health facility as recommended; and finally, a significant relationship to perceived staff attitude, X^2 (1, N= 264) = 18.557, p< 0.001; respondents who perceived staff attitude to be more friendly were likely to report themselves to have sought malaria treatment appropriately.

There was no significant relationship between Malaria treatment seeking behavior and the following respondent variables: age groups (p=0.115), gender (p=0.751), residence status (p=0.831), religion (p=0.785), marital status (p=0.116), respondent college (p=0.419), cost of transportation (p=0.507), service availability (p=0.150), and knowledge of malaria (p=0.497).

Table 2: Characteristics of respondent in the study and relationship with Malaria Treatment Behaviour (N=264)

Variable	Total (N=264)	Malaria Treatment/He[illegible]lth-care Seeking Behaviour ([illegible]) Sought care at health facility, N(%)	Did not seek care at health facility N(%)	X^2	p-value
**Sex**					
Male	135	79(58.5)	56(41.5)	0.101	0.751
Female	129	73(56.6)	56(43.4)		
**Age category**					
18-25	155	82(52.9)	73(47.1)	4.331	0.115
26-33	96	60(62.5)	36(37.5)		
34-41	13	10(76.9)	3(23.1)		
**Continent**					
Africa	188	98 (52.1)	90(47.9)	7.936	**0.005**
Non-Africa	76	54(71.1)	22(28.9)		
**Residential status**					
Off campus	34	19(55.9)	15(44.1)	0.046	0.831
On campus	230	133(57.8)	122(42.2)		
**Religion**					
Christian	204	120(58.8)	84(41.2)	0.818	0.785
Muslim	45	25(55.6)	20(44.4)		
Traditionalist/Atheist	15	8(46.7)	7(53.3)		
**Marital status**					
Single	210	126(60)	84(40)	2.470	0.116
Married	54	26(48.1)	28(51.9)		
**College**					
Health Sciences	61	34(55.7)	27(44.3)	2.826	0.419
Humanities	78	29(37.2)	49(62.8)		
Basic & Allied Science	104	60(57.7)	44(42.3)		
Education	21	9(42.9)	12(57.1)		
**Perceived service accessibility**					
More accessible<30mins	227	123(54.2)	104(45.8)	7.624	**0.006**
Less accessible ≥30mins	37	29 (78.4)	8(21.6)		
**Perceived Waiting time**					
Short (<30mins)	27	4(17.9)	23(82.1)	22.514	**<0.001**
Long (≥30mins)	237	148(62.4)	89(37.6)		
**Treatment Cost**					
More affordable (<15 GHC)	131	115(87.8)	16(12.2)	97.160	**<0.001**
Less affordable≥15GHC	133	37(27.8)	96(72.2)		
**Cost of transport**					
More Affordable <8GHC	114	63(55.3)	51(44.7)	0.439	0.507
Less affordable ≥ 8GHC	150	89(59.3)	61(40.7)		
**Health Insurance**					
No	143	92(64.3)	51(35.7)	5.837	**0.016**
Yes	121	60(49.6)	61(50.4)		
**Perceived service Availability**					
No	198	109(55.1)	89(44.9)	2.688	0.150
Yes	66	43(65.2)	23(34.8)		
**Malaria Knowledge**					

Knowledgeable	229	130(56.8)	99(43.2)	0.461	0.497
Inadequate knowledge	35	22(62.9)	13(37.1)		
**Perceived H/W Attitude**					
Yes(unfriendly)	13	0(0.0)	13(100.0)	18.557	**<0.001**
No(friendly)	251	152(60.6)	99(39.4)		

Predictors of Malaria Treatment (Healthcare) Seeking Behaviour

Respondents who perceived malaria health services accessibility to be less accessible or requiring at least 30 minutes to access services (≥30mins) had at least 7 times the odds of seeking appropriate Malaria treatment health care (aOR = 6.67; 95% CI: 2.35-18.93, p <0.001) as those who perceived service accessibility to be more accessible or within 30 minutes of reach (<30mins) (aOR = 6.67; 95% CI: 2.35-18.93, p <0.001). Those who perceived the waiting time at the facility to be 30 minutes or more (≥30mins) had about 6 times the odds of seeking appropriate health care for Malaria treatment compared to those who perceived the waiting time to be less than 30mins (aOR = 5.94; 95% CI: 2.35-18.93, p= 0.015).

Respondents who thought the cost of malaria treatment at the facility was affordable (less than 15 GHC) had about 20 times the odds of seeking appropriate health care for Malaria treatment compared to those who thought the cost was less affordable (more than 15 GHC), (aOR = 19.88; 95% CI: 9.77-40.46, p<0.001). Respondents aged 34-41 years were approximately 9 times more likely to seek appropriate health care for Malaria treatment than those aged 18-25 years (aOR = 8.83; 95% CI: 1.26-61.94, p=0.028). Respondents aged 26-33 years were about 2.5 times more likely to seek appropriate health care for Malaria treatment than those aged 18-25 years (aOR = 2.50; 95% CI: 1.07-5.48, p=0.034).

.Table 3: Association of Predictors and Malaria Health-care seeking Behaviour (HSB)

	**Binary Logistic regression**			
	**Univariate**		**Multivariable**	
**Variable**	**Crude Odds Ratio [cOR (95% CI)]**	**p-value**	**Adjusted Measure [aOR(95%CI)]**	**p-value**
**SOCIO-DEMOGRAPHIC**				
**Sex**				
Male	1			
Female	0.92(.56-1.51)	0.751		
**Age category**				
18-25	1		1	**0.037**
26-33	1.484(0.88-2.49)	0.137	2.50(1.07-5.48)	**0.034**
34-41	2.96(.786-11.20)	0.108	8.83(1.26-61.94)	**0.028**
**Marital status**				
Single	1	0.118	1	
Married	0.62(.34-1.13)		0.36(.12-1.05)	0.60
**Continent**				
Africa	1	0.560		
Non-Africa	1.09(.82-1.45)			
**ENABLING FACTORS**				
**Perceived service accessibility**				
More accessible<30mins	1		1	

Less accessible ≥30mins	3.07(1.34-6.99)	**0.008**	6.67(2.35-18.93)	**<0.001**
**Perceived Waiting time**				
Short (<30mins)	1		1	
Long (≥30mins)	9.56(3.20-28.55)	**<0.001**	5.94(2.35-18.93)	**0.015**
**Treatment Cost**				
Affordable (<15 GHC)	18.65(9.77-35.58)	**<0.001**	19.88(9.77-40.46)	**<0.001**
Less affordable≥15GHC	1		1	
**Health Insurance**				
No	1			
Yes	1.83(0.12-3.01)	**0.016**		
**Perceived service Availability**				
No	1			
Yes	1.53(0.86-2.73)	0.152		

[4]Crude Odds ratio (cOR) unadjusted

[5]Odds ratios adjusted for for age group, sex, marital status, and continent of origin.

[6]Hosmer and Lemeshow test of goodness of fit for the model was significant at X^2=11.283, df (8) p.value=0.186; Nagelkarke Pseudo R^2= 0.553; Cox & Snell R^2=0.412; log likelihood =219.788;

DISCUSSION

The predictors of Malaria health-care seeking behaviors were investigated in this study of 264 international students at the University of Ghana. The actions taken and factors associated with Malaria health-seeking behaviors of international students at the University of Ghana were demonstrated in this paper. Self-medication was used by 228/264 (86.4%) of respondents as a first line of defense for suspected malaria health seeking behavior. Out of the 228 students with suspected malaria who used self-medication as the first line of treatment, 93/228 (40.8%) purchased antimalarial drugs from a pharmacy or drug shop, 71/228 (31.1%) purchased both antimalarial and analgesic drugs, and 64/228 (28.1%) purchased only analgesic drugs from a medicine and drug selling health facility.

These findings are consistent with those of a study conducted among university students in South Western Nigeria on the prevalence of self-medication, which found that 46.3% of them used antimalarial drugs to treat themselves when they experienced malaria symptoms (22). Furthermore, the findings of this study are consistent with those of a study conducted among university students in Nigeria, where 66% used a variety of medications, including antimalarials, to treat malaria-related symptoms (23). According to a previous study, self-medication for malaria is thought to be more time efficient, less expensive, and easier to obtain than formal health care (24). This type of practice is also expected to occur, particularly when patients believe their disease is in a less severe state, which may occur immediately after taking a few medications (25). It is therefore understandable that a significant proportion of students in this study used self-medication, despite the fact that this inappropriate use of medications has been linked to antimalarial resistance.

In terms of the second line of action, the majority of respondents (57.6%) went to a health facility such as a hospital or clinic for further treatment of malaria-related symptoms, while 38.3% sought health care from pharmacies, among other places. Appropriate malaria treatment health-seeking behavior for malaria as a second-line option after self-treatment failure in this study is higher than the 24.7% among University of Benin students in Nigeria (23). Failure to improve after antimalarial self-prescription is understood to prompt appropriate treatment from a health facility in an attempt to improve (25). This is thought to be

the case when patients who have relied on self-treatment for malaria symptoms realize that they are not improving. Furthermore, because the symptoms of malaria are similar to those of many other diseases, patients are more likely to seek appropriate healthcare services that will substantiate the true causes of the symptoms, thus opting to go to a health facility where better investigations can be carried out if self-treatment fails (26).

More respondents from non-African countries (42.1%) reported using health facility services as a second line of defense during the most recent Malaria treatment action. This finding could be explained by the discovery that students from non-African countries have a higher risk of having severe malaria outcomes than students from African countries (17). Students from non-African countries are more likely to seek malaria health services than African students who perceive a low risk of malaria severity due to their fear of being more harmed by malaria and its complications. Furthermore, African students may encounter traditional malaria treatment methods similar to those found in their home countries. As a result, they are more likely to stick to these practices, which may include the use of herbs that they can obtain. The prospect of becoming ill with malaria-like symptoms may cause non-African students, particularly those from non-endemic countries, to avoid traditional malaria treatment practices that they do not already practice in their home countries.

International students who perceived low cost of appropriate malaria treatment were more likely to seek it than those who thought the cost of appropriate malaria treatment was high. Previous research has shown that university students are more likely to seek healthcare from alternative sources if they believe the cost of care at conventional, including university health facilities, is high (1). The fact that the university hospital provides relatively free primary healthcare to all students may serve as motivation to seek appropriate care without fear of incurring unaffordable costs as revealed in this study.

The study's findings further revealed that international students who perceived they needed at least 30 minutes or more to reach a health facility for appropriate malaria treatment had at least seven times the odds of seeking appropriate Malaria treatment health care compared to those who perceived they needed less than 30 minutes to reach a health facility where appropriate malaria care would be provided. This is an unforeseen finding because one would expect students with perceived easy access to the health facility to have better malaria health seeking behaviors than those with perceived difficult access to the health facility. One possible explanation for this is that students who live further away from health facilities that provide appropriate malaria care will seek appropriate malaria services out of fear of finding it difficult to travel to the health facility if the perceived malaria worsens. Those who believe it is easier to reach health facilities may become complacent and opt for self-medication with the option of going to the health facility later, regardless of the severity of the disease, because they believe it will not be difficult due to the proximity to the health facilities.

This study revealed that respondents who had to wait for at least 30 minutes or more (more than 30 minutes) at the facility had approximately 6 times the odds of seeking appropriate health care for Malaria treatment compared to those who perceived the wait time to be short (less than 30 minutes) (aOR = 5.94; 95% CI: 2.35-18.93, p=0.015). This finding contradicts previous research, which identified long wait times as one of the factors discouraging students from seeking services from university health facilities. It is possible that students in this study who perceived a long waiting time at health facilities sought appropriate malaria treatment because they believed they had to wait longer to receive appropriate malaria diagnostic and treatment services.

Previous research has found that when people have health insurance, they are more likely to seek Malaria diagnosis and treatment from a health facility because they do not have to pay out of pocket(27). Unfortunately, in this study, having health insurance did not influence international students to seek appropriate malaria treatment. This finding is supporting the notion that other factors other than the cost of treatment come into play in order to influence appropriate health seeking behaviours. The findings support a previous study that found that valid health insurance card holders in Ghana used self-medication at times, while others used herbs for malaria treatment(28). In Ghana, despite having valid insurance, more than 15% of insured patients sought care from informal health facilities, citing challenges such as travel time to formal health facilities and waiting times (27). This issue has persisted as a barrier to the delivery of health-care services even to insurance holders in Ghana(29). It will thus require further investigation to be properly addressed.

This study also found that students over the age of 30years were more likely to engage in appropriate malaria treatment seeking behaviors than students under the age of 30 years. This finding is consistent with previous research, which has generally shown that younger students have poorer health seeking behaviors than older students (30,31). Poor health seeking behavior among younger students has previously been attributed to barriers such as high treatment costs, insufficient health information and experience with malaria, high transportation costs to health facilities, complacency that leads to false perceived immunity against malaria, and many others (17). When compared to older students, it is possible that younger students believe their risk of malaria is lower and thus resort to self-management, whereas older students may believe they are at a higher risk of developing severe disease due to their apparent lower immunity. Furthermore, older students are more likely than younger students to not only be living on stipends, but to have more sources of financial survival, which they can use to purchase appropriate healthcare.

Given that all students are required to visit the university medical center as part of their routine health check, university health teams should use these visits to raise awareness about malaria and the options for prevention and treatment among international students. Service providers may also take advantage of this opportunity to identify gaps in health-care needs and how to close those gaps to improve health-seeking behaviors. When implementing malaria treatment behavior improvement measures, younger international students should be prioritized because the causes of poor malaria treatment seeking among them remain unknown. This will entail determining the potential causes of their poor malaria treatment seeking behaviors, as well as what needs to be done to improve the situation.

Study limitations

The findings may not be generalized to the entire population of the University consisting of nationals and international students since the study was limited to only international students.

Conclusion

The purpose of this paper was to investigate malaria treatment seeking behavior and its factors among international students at the University of Ghana. Following the development of malaria-related symptoms, antimalarial drugs were used as first-line therapy. The second course of action was to seek treatment at a health facility (hospital or clinic). Four explanatory variables were found to be independently associated with Malaria Treatment Seeking Behavior among international students: service accessibility, wait time, treatment cost, and respondents' age group. Improved health education about appropriate malaria seeking behaviors, as well as

outreaches to screen and educate students within their halls of residence, may aid in overcoming some of the barriers that have been identified as leading to inappropriate malaria treatment seeking behaviors among international students. The university-based outreach programs can be used to address international students' fears, which may be leading to poor malaria treatment and health seeking behaviors.

AUTHOR CONTRIBUTIONS

ML and PD-G initiated the project. ML conducted the data analysis with considerable input from PG. ML was involved in development of first draft of the manuscript with PG, SPK, and reviewed by PD-G. All authors contributed to the subsequent versions of the manuscript.

Funding

No external funding was received.

Availability of data set and tools

The data set used and analyzed during the study is available from the corresponding author on reasonable request by any individual interested.

Ethics approval, procedures and consent to participate in the study

Ghana Health Service Ethics Review Committee granted ethical approval to conduct the study (Approval No: GHS-ERC023/12/17) (GHS-ERC). Permission was granted by the Dean of the International Program Office, the Dean of Students, and the Senior Hall Tutor for Jubilee Hall and the International Students' Hostel at the University of Ghana where the international students reside. All the participants provided written informed consent. There are no personal identifiers on the tools or analysis.

Consent for publication

Not Applicable.

CONFLICTS OF INTEREST

The authors have no conflicts of interest.

References

1. Afolabi MO, Daropale VO, Irinoye AI, Adegoke AA. Health-seeking behaviour and student perception of health care services in a university community in Nigeria. Ann Ibadan Postgrad Med. 2013;5(5):817–24.

2. Kamau A, Mogeni P, Okiro EA, Snow RW, Bejon P. A systematic review of changing malaria disease burden in sub-Saharan Africa since 2000: Comparing model predictions and empirical observations. BMC Med. 2020;18(1):1–11.

3. Agudelo Higuita NI, White BP, Franco-Paredes C, McGhee MA. An update on prevention of malaria in travelers. Ther Adv Infect Dis. 2021;8:1–17.

4. World Health Organization. World malaria report 2018. Geneva; 2018. Report No.: CC BY-NC-SA 3.0 IGO.

5. World Health Organization. Word Malaria Report 2021. Geneva; 2021.

6. Wambani J, Okoth P. Impact of Malaria Diagnostic Technologies on the Disease Burden in the Sub-Saharan Africa. J Trop Med. 2022;2022.

7. Steketee RW, Choi M, Linn A, Florey L, Murphy M, Panjabi R. Perspective piece world malaria day 2021: Commemorating 15 Years of Contribution by the United States President's Malaria Initiative. Am J Trop Med Hyg. 2021;104(6):1955–9.

8. Awine T, Malm K, Bart-Plange C, Silal SP. Towards malaria control and elimination in Ghana: Challenges and decision making tools to guide planning. Glob Health Action [Internet]. 2017;10(1). Available from: https://doi.org/10.1080/16549716.2017.1381471

9. Shretta R, Silal SP, Malm K, Mohammed W, Narh J, Piccinini D, et al. Estimating the risk of declining funding for malaria in Ghana: The case for continued investment in the malaria response. Malar J [Internet]. 2020;19(1):1–15. Available from: https://doi.org/10.1186/s12936-020-03267-9

10. Orok AB, Ajibaye O, Aina OO, Iboma G, Adagyo Oboshi S, Iwalokun B. Malaria interventions and control programes in Sub-Saharan Africa: A narrative review. Cogent Med [Internet]. 2021;8(1):1940639. Available from: https://doi.org/10.1080/2331205X.2021.1940639

11. Bruxvoort KJ, Leurent B, Chandler CIR, Ansah EK, Baiden F, Bjorkman A, et al. The impact of introducing malaria rapid diagnostic tests on fever case management: a synthesis of ten studies from the ACT consortium. Am J Trop Med Hyg. 2017;97(4):1170–9.

12. Orish VN, Maalman RSE, Donkor OY, Ceruantes BYH, Osei E, Amu H, et al. Assessing health-seeking behaviour and malaria prevention practices among communities in four districts of the Volta Region of Ghana. Malar J [Internet]. 2021;20(1):1–13. Available from: https://doi.org/10.1186/s12936-021-03986-7

13. Tizifa TA, Kabaghe AN, McCann RS, van den Berg H, Van Vugt M, Phiri KS. Prevention Efforts for Malaria. Curr Trop Med Reports. 2018;5(1):41–50.

14. World Health Organization. Malaria. In: International Travel and Health. 2020th ed. Geneva: World Health Organization; 2020. p. 260.

15. Duguma T, Tekalign E, Muleta D, Simieneh A. Malaria prevalence and risk factors among patients visiting Mizan Tepi University Teaching Hospital, Southwest Ethiopia. PLoS One [Internet]. 2022;17(7):e0271771. Available from: http://dx.doi.org/10.1371/journal.pone.0271771

16. Askling HH, Nilsson J, Tegnell A, Janzon R, Ekdahl K. Malaria risk in travelers. Emerg Infect Dis. 2005;11(3):436–41.

17. Heggenhougen HK behavioural and social aspects of malaria and its control, Hackethal V, Vivek P. The behavioural and social aspects of malaria and its control: An introduction and annotated bibliography. TDR/STR/SEB/VOL/03.1. 2003.

18. OXFAM. End of Project Evaluation: Mother and motherhood through advocacy and actions. 2019. p. 9–25.

19. The University of Ghana. Information brief [Internet]. Accra: The University of Ghana; 2017. Available from: https://www.ug.edu.gh/pad/sites/pad/files/u6/paddocs/INFO BRIEF.compressed.pdf

20. The Univerisity of Ghana. Facts and figures [Internet]. 2016. Available from: https://www.ug.edu.gh/pad/sites/pad/files/u6/paddocs/INFO BRIEF.compressed.pdf

21. Leslie K. Survey sampling. New York: John Wiley and Sons; 1965.

22. Osemene KP, Lamikanra A. A study of the prevalence of self-medication practice among university students in southwestern Nigeria. Trop J Pharm Res. 2012;11(4):683–9.

23. Adeyemo F. O, Okpala P. U, Oyana EN, Imoukhuede MO. Malaria infection amongst students of the University of Benin, Edo State, Nigeria. Int J Recent Sci Res. 2014;5(9):1529–32.

24. McCombie SC. Self-treatment for malaria: The evidence and methodological issues. Health Policy Plan. 2002;17(4):333–44.

25. Deressa W. Treatment-seeking behaviour for febrile illness in an area of seasonal malaria transmission in rural Ethiopia. Malar J. 2007;6(49):1–7.

26. Clark IA, Alleva LM, Mills AC, Cowden WB, Ev CLINMIR. Pathogenesis of Malaria and Clinically Similar Conditions. 2004;17(3):509–39.

27. Fenny AP, Asante FA, Enemark U, Hansen KS. Malaria care seeking behavior of individuals in Ghana under the NHIS: Are we back to the use of informal care? Global health. BMC Public Health. 2015;15(1):1–8.

28. Dalaba MA, Akweongo P, Aborigo R, Azongo DK, Asaana P, Atuguba F, et al. Does the national health insurance scheme in Ghana reduce household cost of treating malaria in the Kassena-Nankana districts? 2014;9716(May).

29. Aidam K. Implementation of the National Health Insurance Scheme (NHIS) in Ghana : Lessons for South Africa and Low- and Middle-Income Countries. Risk Manag Healthc Policy. 2020;13:1879–904.

30. Kahi H, Rizk GA, Hlais S. Health-care-seeking behaviour among university students in Lebanon. East Mediterr Heal J. 2012;18(6):598–606.

31. Silvestri-elmore A. Healthcare-seeking behaviours in college students and young adults : a review. J Res Nurs. 2021;26(4):320–38.
