# Supplementary material for: Malaria treatment health seeking behaviors among international students at the University of Ghana Legon
Source: PLoS One. 2023 Oct 26;18(10):e0276412. doi: 10.1371/journal.pone.0276412 (PMC10602253; doi:10.1371/journal.pone.0276412)
Supplement: Attachment — Submitted filename: Comments to author plos one.docx [file pone.0276412.s002.docx]

Comments to author

1- Malaria treatment health-seeking behaviors among international students at the University of Ghana Legon, Accra

The title is not clear, is to better to avoid the word health from it , in-order to make it smart

2- Abstract- not catchy and contains unnecessary results

Introduction- doesn’t shows a gap in line with the study necessities

Method – doesn’t highlights every aspect , like sampling procedure, stastical decision cut points

Results- not important to mention unfinished results ( bi-variable results)

Conclusion – no in lines with the result.

3- Check appropriate contents o title page ( manuscript organization) available at <https://journals.plos.org/plosone/s/submission-guidelines>
4- Introduction part- paragraph 3… Error! Reference source not found.,Error! Reference source not found.. avoid and make necessary arrangements
5- Method part- rename as methods and materials

Study design….definition of the approach should be putted in operational definition

Study setting- say something about the malaria service

Population--- redefine your population, source and study population …840 students are not your study population

Study variables and measurements- please clearly put operational definition and variables or put our variables( dependent and independent variables clearly)- avid unnecessary detail eg resident( we can see it in result part)

Data quality assurance- not said

Data management and analysis- is bivariate or bi-variable,

Avoid unnecessary details , it difficult to understand

6- Result

Age- put important description (used in model) in result part

Better to put only the results--- avoid unnecessary descriptions ---We investigated the respondents' immediate action in terms of seeking treatment as soon as they suspected they had malaria-like symptoms. Table 1 shows detailed cross-tabulations of actions taken to seek Malaria treatment. Five patterns of malaria treatment seeking behavior emerged. These included health seeking behaviors toward orthodox care, such as treatment being facilitated by purchasing medicines from a pharmacy for either antimalarial treatment only or antimalarial and analgesics, or for some purchasing only analgesics. Other non-orthodox malaria treatment seeking behaviors included seeking care at a health facility or using alternative treatments such as herbs, prayers, and warm baths… either put this issue in methods and materials part

Needs table citation – after narration of General characteristics of the respondents

Predictors --- from where u reported AOR of reference variables, which u put it similar with … Respondents who perceived malaria health services accessibility to be less accessible or requiring at least 30 minutes to access services (≥30mins) had at least 7 times the odds of seeking appropriate Malaria treatment health care (aOR = 6.67; 95% CI: 2.35-18.93, p <0.001) as those who perceived service accessibility to be more accessible or within 30 minutes of reach (<30mins) (aOR = 6.67; 95% CI: 2.3518.93, p <0.001).

Unless otherwise you will discuss the each results you don’t need to describe it separately…..the bivariable results..merge it with multivariable table and finish it with two –three sentence

Outcome measurement is putted for your dependent variable but not summarized in result part…simply putted as descriptive stastical your first objective ..needs to have overall result based on your operational definition.

7- Discussion--- similar with results/repetition of result ….not discussed vigorously different studies are conducted on your title in different pats of Africa so discuss it in detail. Especially on important findings(pertinent) .

Needs major revision - should inline with your study objective

8- Conclusion- not in-line with the findings. Your objective is to investigate malaria treatment seeking behavior and its factors among international student… but the conclusion is different and full of less important issues.
