# Supplementary material for: Malaria treatment health seeking behaviors among international students at the University of Ghana Legon
Source: PLoS One. 2023 Oct 26;18(10):e0276412. doi: 10.1371/journal.pone.0276412 (PMC10602253; doi:10.1371/journal.pone.0276412)
Supplement: Attachment — Submitted filename: Response to Reviewers.docx [file pone.0276412.s004.docx]

A rebuttal letter that responds to each point raised by the academic editor and reviewer(s)

**Tittle**:Malaria treatment seeking behaviors among international students at the University of Ghana Legon, Accra

**Response to reviewer’s comments**

| **Comment** | **Response** | **Pages or sentences where the changes have been effected in the revised manuscript** |
| --- | --- | --- |
| **Tittle page** | | |
| 1. The title is not clear, is to better to avoid the word health from it , in-order to make it smart | The word health from the tittle has been eliminated from all pages where it was used to refer to Malaria Health Seeking behaviour to malaria treatment seeking behaviour | Pg.1 and through out the document where it has been used. |
| 2. Abstract- not catchy and contains unnecessary results   Introduction- doesn’t shows a gap in line with the study necessities  Method – doesn’t highlights every aspect , like sampling procedure, statistical decision cut points  Results- not important to mention unfinished results ( bi-variable results)  Conclusion – not in lines with the result | These have been corrected  Sampling method as systematic sampling and a level of 0.2 at bi-variable and 0.05 at multi-variable analysis at 95% CI  Bi-variable results that are incomplete removed  Conclusion has been revised | Pg 2 |
| The use of appropriate is too much in this section  The Ghana Health Service has Standard Treatment Guidelines for treating and testing malaria. Read this document and incorporate it in your work | This has been reduced in the entire document  This has been in-cooperated where they emphasize 3Ts in management of malaria |  |
| Who/which institution should implement this recommendation? I suggest you have a conversation with the the UG students clinic, They have improved their services significantly and you can have ideas on how to use their systems to inform your recommendations | This has been improved by recommending the international house together with the university of Ghana clinic to support in increasing awareness about the appropriate malaria treatment seeking behaviour emphasizing the 3Ts |  |
| Indicate why this population was targeted | This has been included, indicating that most of them come from low malaria endemic area. |  |
| Check appropriate contents of title page ( manuscript organization) available at <https://journals.plos.org/plosone/s/submission-guidelines> | Checked for appropriate content of tittle page . Manuscript has been formatted as per the guidelines | All pages of the manuscript |
| **Introduction** | | |
| Introduction part- paragraph 3… Error! Reference source not found.,Error! Reference source not found.. avoid and make necessary arrangements | This has been corrected | Pg 3 |
| Healthcare seeking behaviour is any action or inaction taken by individuals who believe they have a health problem or are ill in order to find proper medication (move this elsewhere and start with sentence 2) | Started with sentence two and this was moved to the same subsection of the introduction | Sentence 12 Pg 3 |
| Ineffective malaria management due to incorrect decisions may result in delayed diagnosis and treatment,  increasing the risk of severe disease and death **Error! Reference source not found.**,**Error! Reference**  **source not found.** | This has been deleted from the manuscript | Sentence 13,Pg 3 |
| The study should be contextualized within the UG health system and the students clinic services. This is the first point of call for all UG students | This has been contextualized within the University of Ghana health system | Pg 3 |
| **Methods and materials** | | |
| Method part- rename as methods and materials | Renamed as Methods and materials | Pg.4 |
| Please correct the use of upper case and lower case letters. Be consistent | These have been corrected | Through out the document as per the guidelines |
| Use invited | Used the word invited instead of summoned under the section of data collection and instruments | Pg 4 |
| Study design….definition of the approach should be putted in operational definition | This has been removed from the study design section |  |
| Study setting- say something about the malaria service | This has been revised | Pg. 3 |
| Population--- redefine your population, source and study population …840 students are not your study population | This was corrected to include all students as of July 2018 at the university of Ghana coming from other countries and registered by the international house | Pg .4 |
| Study variables and measurements- please clearly put operational definition and variables or put our variables( dependent and independent variables clearly)- avid unnecessary detail e.g resident( we can see it in result part) | These have been separated and given sub-headings | Pg 5 |
| Where malaria testing and management is done for cardinal malaria symptoms such as fever, headache, and joint pains | This has been deleted from the manuscript under the section of study variables and measurements | Pg 4 |
| More affordable (<15 Ghana Cedis (GHC) and less affordable | This has been deleted | Pg 5 |
| Repititve;Health insurance coverage (yes or no). | Deleted from the document | Pg.5 |
| Data were checked for completeness and consistency, coded, and entered into a predesigned excel template before being exported to IBM's SPSS statistical software version 22 (V22) for analysis. At the univariate level, descriptive statistics were used to organize and summarize the data as frequencies, proportions for categorical variables, and mean values for continuous variables. In bivariate analysis, the X2  ) Pearson Chi-square statistics ( were used | Deleted from the document under section of data management | Pg.5 |
| Data quality assurance- not said | This has been added | Pg 5 |
| Data management and analysis- is bi-variate or bi-variable, | This has been changed to bi-variable | Pg 6 |
| Avoid unnecessary details , it difficult to understand | This has been addressed |  |
|  |  |  |
| **Results** | | |
| Age- put important description (used in model) in result part | This has been addressed | Pg 7 |
| Better to put only the results--- avoid unnecessary descriptions ---We investigated the respondents' immediate action in terms of seeking treatment as soon as they suspected they had malaria-like symptoms. Table 1 shows detailed cross-tabulations of actions taken to seek Malaria treatment. Five patterns of malaria treatment seeking behavior emerged. These included health seeking behaviors toward orthodox care, such as treatment being facilitated by purchasing medicines from a pharmacy for either antimalarial treatment only or antimalarial and analgesics, or for some purchasing only analgesics. Other non-orthodox malaria treatment seeking behaviors included seeking care at a health facility or using alternative treatments such as herbs, prayers, and warm baths… either put this issue in methods and materials part | This has been placed in the methods and materials section. Transferred to pg 5 | Pg.7 |
| Needs table citation – after narration of General characteristics of the respondents | This has been cited after narration | Pg. 8 |
| Please correct this. seeking care at a health facility is standard | This has been corrected | Pg. 8 |
| What is recommended by the STG of the GHS | This has been corrected as if one gets malaria related symptoms , they should get tested, treated if positive ad tracked | Pg.8 |
| Which HSB are did you use for this analysis? First action or second action. The sample size varies from the table presented above where only 10 respondents went to a health facility.  If only 10 respondents went to a health facility this will affect the bivariate and multivariate analysis | We used second line action including those | Pg 9 |
| Predictors --- from where u reported AOR of reference variables, which u put it similar with … Respondents who perceived malaria health services accessibility to be less accessible or requiring at least 30 minutes to access services (≥30mins) had at least 7 times the odds of seeking appropriate Malaria treatment health care (aOR = 6.67; 95% CI: 2.35-18.93, p <0.001) as those who perceived service accessibility to be more accessible or within 30 minutes of reach (<30mins) (aOR = 6.67; 95% CI: 2.3518.93, p <0.001). | This has been corrected | Pg 10 |
| Unless otherwise you will discuss the each results you don’t need to describe it separately…..the bivariable results..merge it with multivariable table and finish it with two –three sentence | This has been revised | Pg. 8 |
| Outcome measurement is putted for your dependent variable but not summarized in result part…simply putted as descriptive statistical your first objective ..needs to have overall result based on your operational definition |  | Pg. 8 |
|  |  |  |
| **Discussion** | | |
| Discussion--- similar with results/repetition of result ….not discussed vigorously different studies are conducted on your title in different pats of Africa so discuss it in detail. Especially on important findings(pertinent) .Needs major revision - should inline with your study objective | This has been revised | Pg 11 |
| The repetition of results in this section is too much | This has been addressed | Pg. 11 |
| **Conclusion** | | |
| Conclusion- not in-line with the findings. Your objective is to investigate malaria treatment seeking behavior and its factors among international student… but the conclusion is different and full of less important issues | This has been improved | Pg. 14 |
| Since appropriate sampling methods were used, I think this is not the major limitation of the study.  The authors should consider recall bias as a limitation instead | Recall bias has been considered as the main limitation of this study | Pg. 14 |

By Mathias Lwenge

Date: 24^th^ December 2022
