# Supplementary material for: Malaria treatment health seeking behaviors among international students at the University of Ghana Legon
Source: PLoS One. 2023 Oct 26;18(10):e0276412. doi: 10.1371/journal.pone.0276412 (PMC10602253; doi:10.1371/journal.pone.0276412)
Supplement: Attachment — Submitted filename: My comments.docx [file pone.0276412.s005.docx]

**My comments regarding the amended manuscript:**

1. I note an improvement in the revised version of the manuscript.
2. Still, there are numerous typographical errors that need to be updated or fixed.
3. Issues concerning grammar use should be corrected.
4. The language ought to get better as well.
