# Supplementary material for: Malaria treatment health seeking behaviors among international students at the University of Ghana Legon
Source: PLoS One. 2023 Oct 26;18(10):e0276412. doi: 10.1371/journal.pone.0276412 (PMC10602253; doi:10.1371/journal.pone.0276412)
Supplement: Attachment — Submitted filename: Comments to malaria.docx [file pone.0276412.s006.docx]

1. Comments….Unless otherwise you will discuss the each results you don’t need to describe it separately…..the bivariable results..merge it with multivariable table and finish it with two –three sentence

Outcome measurement is putted for your dependent variable but not summarized in result part…simply putted as descriptive stastical your first objective ..needs to have overall result based on your operational definition.

Not corrected – still there is separate table also there is wrong term in table 3 which is Univariable

2. Conclusion- not in-line with the findings---still not corrected
