# Supplementary material for: Malaria treatment health seeking behaviors among international students at the University of Ghana Legon
Source: PLoS One. 2023 Oct 26;18(10):e0276412. doi: 10.1371/journal.pone.0276412 (PMC10602253; doi:10.1371/journal.pone.0276412)
Supplement: Attachment — Submitted filename: Response to Reviewers october 2023.docx [file pone.0276412.s007.docx]

A rebuttal letter that responds to each point raised by the academic editor and reviewer(s)

**Tittle**: Malaria treatment seeking behaviors among international students at the University of Ghana Legon, Accra

**Response to reviewer’s comments**

| **Comment** | **Response** | **Pages or sentences where the changes have been effected in the revised manuscript** |
| --- | --- | --- |
| **Tittle page** | | |
| Unless otherwise you will discuss the each results you don’t need to describe it separately…..the bivariable results. Merge it with multivariable table and finish it with two –three sentence | The methodology section has been revised to eliminate the univariatedescription. The multivaribles have been merged in table 2 | Pg,6 and 9 |
| Outcome measurement is putted for your dependent variable but not summarized in result part…simply putted as descriptive stastical your first objective needs to have overall result based on your operational definition | This has been included under malaria treatment health seeking behaviours with 35.2% purchasing antimalrial drugs alone. | Pg 7 |
| Not corrected – still there is separate table also there is wrong term in table 3 which is Univariable | The table 3 has been removed by merging it with table 2 | Pg 8 and 9 |
| Conclusion- not in-line with the findings---still not corrected | These have been corrected | Pg 12 |
| Still, there are numerous typographical errors that need to be updated or fixed. | These have been corrected through proof reading by the authors | All pages of the manuscript |
| Issues concerning grammar use should be corrected. | These have been corrected through proof reading by the authors | All pages of the manuscript |
| The language ought to get better as well | These have been corrected through proof reading by the authors | All pages of the manuscript |

By Mathias Lwenge

Date: 2^nd^ September 2023
